# Investigating the Rhizosphere Fungal Communities of Healthy and Root-Rot-Infected *Lycium barbarum* in the Tsaidam Basin, China

**DOI:** 10.3390/microorganisms12122447

**Published:** 2024-11-28

**Authors:** Guozhen Duan, Guanghui Fan, Jianling Li, Min Liu, Youchao Qi

**Affiliations:** 1 Academy of Agriculture and Forestry Sciences, Qinghai University, Xining 810016, China; 18848110959@163.com (G.D.); qhfgh@163.com (G.F.); lijianlingmail@163.com (J.L.); 2 Qinghai Plateau Key Laboratory of Tree Genetics and Breeding, Xining 810016, China; 3 College of Agricultural and Forestry Engineering and Planning, Tongren University, Tongren 554300, China; liumin19890110@yeah.net; 4 College of Agriculture and Animal Husbandry, Qinghai University, Xining 810016, China

**Keywords:** *Lycium barbarum*, rhizosphere soil, root rot, fungal community composition, soil chemical properties

## Abstract

*Lycium barbarum* is a plant of considerable economic importance in China. However, root rot poses a significant threat to its yield and quality, leading to substantial economic losses. The disparities in rhizosphere soil fungal communities between healthy and root-rot-affected *L. barbarum* have not been thoroughly explored. Delving into the dynamics between these fungal communities and the onset of root rot may provide pivotal insights for the biological control of this disease in *L. barbarum*, as well as aid in identifying fungi associated with the condition. In this study, we utilized rhizosphere soil samples from Ningqi No. 1, a distinguished cultivar of *L. barbarum*, as our experimental material. We assessed the composition and diversity of fungal communities in both diseased (D) and healthy (H) samples using Illumina MiSeq sequencing technology. The study’s findings revealed that the mean concentrations of total nitrogen (TN) and soil organic matter (SOM) were significantly higher in the healthy specimens when contrasted with the diseased ones, while the pH levels were notably increased in the latter group. Additionally, the alpha-diversity of fungal communities was observed to be greater within the healthy samples as opposed to the diseased samples. Marked distinctions in fungal diversity were discerned between the healthy (H) and diseased (D) samples. Ascomycota was identified as the predominant fungal phylum in both groups. In the healthy samples, beneficial fungi such as *Plectosphaerella* and *Mortierella* were prevalent, in contrast to the diseased samples, the relative abundances of *Embellisia* and *Alternaria* demonstrated remarkable increases of 89.59% and 87.41%, respectively. Non-metric Multidimensional Scaling (NMDS) illustrated clear distinctions in the composition of fungal communities between the healthy and diseased samples. Redundancy Analysis (RDA) indicated total nitrogen (TN), organic matter (SOM), total phosphorus (TP), Available Potassium (AK), pH, and Total Potassium (TK). Notably, pH showed a stronger correlation with the diseased samples, while TN and SOM were more significantly associated with the healthy samples.

## 1. Introduction

The genus *Lycium*, commonly known as wolfberry or Goji, belongs to the Solanaceae family and serves both medicinal and nutritional purposes [1]. Research has indicated that Goji is rich in polysaccharides, betaine, carotenoids, and other bioactive compounds that exhibit significant immunomodulatory effects and the ability to inhibit the growth and mutation of cancer cells [2]. This genus is widely distributed across various regions of the world, including China, the United States, South Korea, Japan, South America, and several other countries, and is recognized for its high nutritional value as well as its salt and drought resistance [3].

In China, *Lycium* is extensively distributed, encompassing seven species and three taxonomic varieties. *L. barbarum* is the most prevalent due to its robust environmental adaptability, primarily found in northern Sichuan, Inner Mongolia, Shaanxi, Gansu, Ningxia, Qinghai, and Xinjiang [4]. Notably, Qinghai province celebrated as the “roof of the world”, is situated in the northeastern segment of the Qinghai-Tibet Plateau and boasts the densest continuous cultivation of *L. barbarum*, reaching a peak occupation of 49,300 hectares [5]. However, as the duration and area of continuous cropping increase, soil-borne diseases such as root rot have become increasingly common in *L. barbarum*. Root rot results in varying degrees of decay in the roots and rhizomes, exposing the xylem and leading to the formation of dark brown vascular bundles. This condition is often referred to as "plant cancer" due to its difficult detection and treatment [6]. Currently, the incidence of root rot in the primary production areas of *L. barbarum* in Qinghai has reached approximately 20%, with an annual increase of 1000 hectares. The incidence rate in local plots can be as high as 53.2% [7]. This situation has significantly impacted the yield and economic returns for farmers, posing a substantial threat to the growth of the industry.

It is widely recognized that root rot, a prevalent soil-borne disease, is instigated by pathogens resident in the soil. According to Koch’s postulates, the primary pathogenic agents responsible for root rot in *L. barbarum* have been identified as multiple species of *Fusarium* spp. (including *F. acuminatum*, *F. oxysporum*, *F. solani*, *F. concolor*, *F. moniliforme*, *F. equiseti*, *F. incarnatum*, and *R. solani*) [8]. The current control measures predominantly rely on chemical agents, which have proven ineffective and have led to the development of pathogen resistance, exacerbating the condition. Moreover, the excessive application of chemical pesticides contributes to environmental pollution and excessive pesticide residues in products, adversely affecting human health and product exports. Increasing evidence implicates an imbalance in the rhizospheric soil microecology as a significant factor triggering root rot diseases, making the study of rhizospheric soil microecology a focal point for the prevention and control of root rot and a breakthrough in resolving the issue [9]. It has been suggested that the accumulation of macronutrients in the soil becomes insufficient to support continuous monocropping of *L. barbarum* for over a decade [10]. To ensure healthy plant growth, it is essential to augment soil compound fertilizer with fertilizers to enrich necessary nutrients. Nevertheless, the focus on micronutrients and continuous mono-cropping often overlooks the potential of root systems to effectively mobilize and absorb soil nutrients, resulting in imbalances in the soil microecology [11,12]. Intensive research has demonstrated that as the duration of plant cultivation increases, the diversity of bacterial species in the soil significantly declines, leading to a simplified community structure. Consequently, the soil microflora transitions from a bacterial-dominant to a fungal-dominant composition [13].

Fungal communities, as a vital group within soil microorganisms, participate in the decomposition of animal and plant remains, becoming an indispensable force in the nitrogen and carbon cycles of the soil [14]. Particularly in the early stages of plant organic matter decomposition, fungi are more active than bacteria and actinomycetes. The transformation of soil organic matter and other nutrients is influenced by the composition and quantity of soil fungal species [15]. Studies indicate a close relationship between soil fungal diversity and the occurrence of soil-borne diseases, as well as plant health. Li Xueping’s research found that the occurrence of root rot diseases in Highland barley altered the fungal community structure in the rhizosphere soil, with more severe disease corresponding to a greater difference in the microbial community structure compared to healthy plants [16]. A structurally rich and diverse soil microbial community can inhibit soil-borne diseases, resulting in milder occurrences; conversely, a less diverse community may lead to more severe diseases. However, as Gustavo Santoyo discussed in his review, the interaction between plants and their microbiome can alter with changes in nutritional demands and environmental conditions. Under certain circumstances, the balance between plant and microbiome interactions can be disrupted, causing previously non-pathogenic normal flora to become pathogenic [17]. Conversely, under stress such as drought, microorganisms typically possessing pathogenicity may transform into mutualistic relationships, aiding plants in adapting to stress. This adaptability highlights the flexibility and plasticity of the relationship between plants and microorganisms [18]. Yet, there remains no exploration into the changes in the fungal community structure of the healthy *L. barbarum* plants in the Qinghai-Tibet Plateau’s Qaidam Basin after the plants have become diseased or the impact of the environment on the fungal structure in this cultivation area.

Therefore, this study employed *L. barbarum* samples from the Qaidam Basin of Qinghai province to investigate the alterations in rhizosphere soil fungal community diversity and composition between healthy and root-rot-infected plants. The detection and comparison of these changes were achieved through internal transcribed spacer (ITS) ribosomal rRNA sequencing technology. The principal objectives of this research were to comprehensively analyze and compare the fungal diversity present in the rhizosphere soil of healthy and unhealthy plants and to examine the relationship between fungal communities, root rot, and soil. This study may contribute to further insights into the mechanisms by which the microbiome functions in the health of plants and diseased specimens. We anticipate that by strategically modulating microbial communities, we can enhance crop resistance to diseases, thereby achieving a green and sustainable agricultural production model.

## 2. Materials and Methods

### 2.1. Study Site

**Figure 1:**
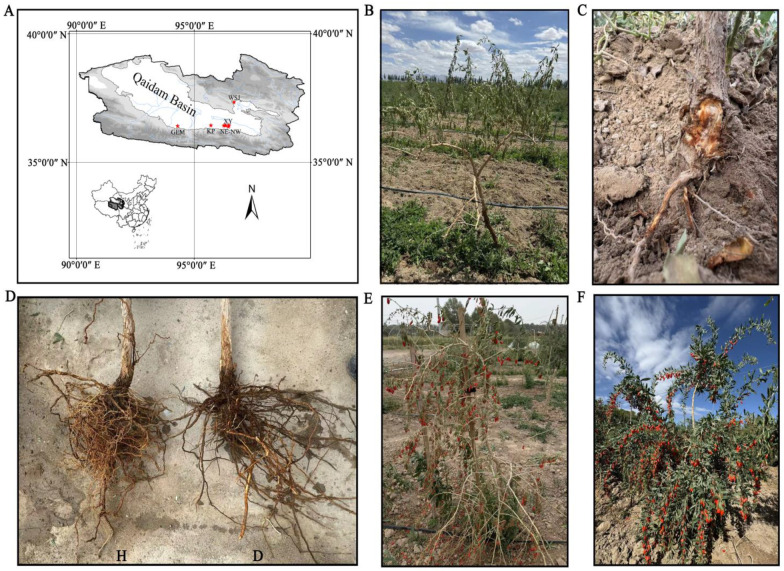
(**A**) represents the sampling sites of *L. barbarum*; (**B**) depicts leaves exhibiting wilted conditions; (**C**) illustrates the stems in contact with the ground, which have been excavated to inspect for swelling; (**D**) contrasts the root systems of healthy plants (H) with those of infected plants (D); (**E**) shows the post-fruiting performance of the infected plants; and (**F**) displays the outcomes of the healthy plants.

The research site is situated in the northwestern region of the Qaidam Basin, within the Haixi Mongolian and Tibetan Autonomous Prefecture of Qinghai Province (refer to Figure 1A). The area is distinguished by its sandy soil composition and low nutrient content. It receives a mere 15 mm of annual precipitation, with an average temperature of 5 °C, experiencing temperature fluctuations as wide as 12 °C [19]. The indigenous flora in the study area comprises *Haloxylon ammodendron*, *L. barbarum*, *Nitraria tangutorum*, *L. ruthenicum*, and *Phragmites australis*. In the northwest of the Qaidam Basin, *L. barbarum* is extensively cultivated as a pivotal economic forest species.

### 2.2. Sample Processing

As the duration of cultivation has lengthened and the planted area has expanded, instances of root rot have become increasingly prevalent in numerous *L. barbarum* plantations. An investigation into the occurrence of root rot in these areas within the Qaidam Basin was conducted in late July 2021. Plants exhibiting severe symptoms of root rot were selectively chosen for the collection of rhizosphere soil, with the soil from healthy plants serving as controls for comparison in the same plot as the diseased plants. In determining the health status of *L. barbarum* plants, the diagnostic process initially involves assessing whether the foliage above ground displays signs of wilting (Figure 1B). Wilting is a preliminary indication of disease. Subsequently, the stems in contact with the ground are excavated to examine for swelling (Figure 1C). Further, the root systems are carefully unearthed to assess the reduction in fine roots and to check for discoloration or decay in the thicker roots (Figure 1D). These symptoms confirm the presence of root rot. Conversely, plants free from these symptoms are considered healthy. Rhizosphere soil is meticulously collected by scraping the soil from the roots with forceps and then passing through a 2-mm sieve. For this study, rhizosphere soils from 51 diseased and 51 healthy *L. barbarum* plants were sampled, respectively (Table 1). At each of the nine sampling sites, three healthy soil samples and three diseased soil samples were individually collected in sterile plastic bags to create a single mixed healthy soil sample and a single mixed diseased soil sample. Due to the severe root rot in the NE plot, it was not feasible to identify more representative specimens. Consequently, only six standard plants were collected and pooled to form two composite samples. A total of 34 samples were prepared, comprising 17 diseased plants (Figure 1E), denoted uniformly as “D”, and 17 healthy plants (Figure 1F), labeled uniformly as “H”. All samples were transported back to the laboratory under liquid nitrogen and subsequently stored at −80 °C for future high-throughput sequencing.

### 2.3. Determination of Soil Physicochemical Properties

The total nitrogen (TN) was measured by the semi-micro Kelvin method [20]; Soil-determination of total phosphorus (TP) was determined by alkali fusion-Mo-Sb Anti spectrophotometric method [21]; The total potassium (TK) was acquired by flame photometer method [22]; The available phosphorus (AP) was determined by NaHCO_3_ extraction molybdenum-antimony colorimetry; the available nitrogen (AN) was determined by the alkali difusion method; Available potassium (AK) was determined with the fame photometric method [23]. The soil OM content (SOM) was measured by the potassium dichromate external heating method [24]. The pH value of the soil was measured with a pH meter/potentiometer under the soil: water ratio of 1:2.5 [25].

### 2.4. DNA Extraction, PCR Amplification and ITS2 Sequencing

DNA was extracted from the various samples utilizing the E.Z.N.A. Stool DNA Kit (D4015, Omega Inc., Norwalk, CT, USA) in accordance with the manufacturer’s guidelines. Nuclease-free water served as the negative control. The total DNA was eluted in 50 μL of elution buffer and stored at −80 °C until PCR analysis, which was conducted by LC-Bio Technology Co., Ltd. (Hangzhou, China). 

The ITS2 region of the eukaryotic small-subunit rRNA gene was amplified using modified versions of the primers ITS1FI2 (5′-GTGARTCATCGAATCTTTG-3′) and ITS2 (5′-TCCTCCGCTTATTGATATGC-3′) [26]. The 5′ ends of the primers were labeled with specific barcodes corresponding to each sample. PCR amplification was carried out in a total reaction volume of 25 μL, which included 25 ng of template DNA, 12.5 μL of PCR premix, 2.5 μL of each primer, and PCR-grade water to complete the volume. The PCR conditions for amplifying the fungi (ITS2) variable region consisted of an initial denaturation step at 98 °C for 30 s, followed by 32 cycles of denaturation at 98 °C for 10 s, annealing at 54 °C for 30 s, and extension at 72 °C for 45 s, concluding with a final extension at 72 °C for 10 min. The PCR products were verified through 2% agarose gel electrophoresis. Throughout the DNA extraction process, ultrapure water was utilized as a negative control to mitigate the risk of false-positive PCR results. The PCR products were purified using AMPure XT beads (Beckman Coulter Genomics, Danvers, MA, USA) and subsequently quantified using Qubit (Invitrogen, Waltham, MA, USA). The amplicon pools were prepared for sequencing, and the size and quantity of the amplicon library were evaluated using an Agilent 2100 Bioanalyzer (Agilent, Santa Clara, CA, USA) and the Library Quantification Kit for Illumina (Kapa Biosciences, Woburn, MA, USA), respectively. The libraries were sequenced on the NovaSeq PE250 platform, facilitated by LC-Bio (Hangzhou, China).

### 2.5. Data Analysis

Paired-end reads were assigned to samples based on their unique barcodes and truncated by removing the barcode and primer sequences. The paired-end reads were merged using PEAR software (version 0.9.6) [27]. Quality filtering of the raw reads was executed under specific filtering criteria to yield high-quality clean tags using fqtrim (version 0.94) [28]. Chimeric sequences were eliminated using VSEARCH software (version 2.3.4) [29]. Following dereplication with DADA2 [30], the feature sequence was obtained at a 99% similarity threshold. The alpha diversity and β-diversity metrics were computed using QIIME 2 2019.457 with slight modification according to the official tutorials (https://docs.qiime2.org/2019.4/tutorials/ accessed on 20 November 2024) [31], whereby the same number of sequences was randomly extracted by reducing the sequence count to that of the sample with the fewest sequences. Taxonomic composition was determined based on relative abundances. Beta diversity metrics (Non-metric Multidimensional Scaling, NMDS): Jaccard distance and Bray–Curtis dissimilarity. RDA was used to reveal the relationships between microbiota and soil properties. This analysis was carried out with “thevegan” package in R (v 3.2.0). FUNGuild, which was used to predict the structure of soil fungal communities in the rhizosphere based on OTU data [32]. LEfSe was used to elucidate the biomarkers in each group [33]. The network diagram was drawn using Cytoscape (v 3.10.2) [34]. Venn diagrams were generated using PRIMER 7 [35]. Both ANOVA and Spearman’s rank correlations between abundant genera and soil properties were performed in SPSS 22.0 (SPSS Inc., Chicago, IL, USA). All statistical tests considered *p* < 0.05 as the threshold for significance by the Student’s t-test at *p* < 0.05. H: healthy samples; D: diseased samples.

## 3. Results

### 3.1. Chemical Properties of Soil

**Figure 2:**
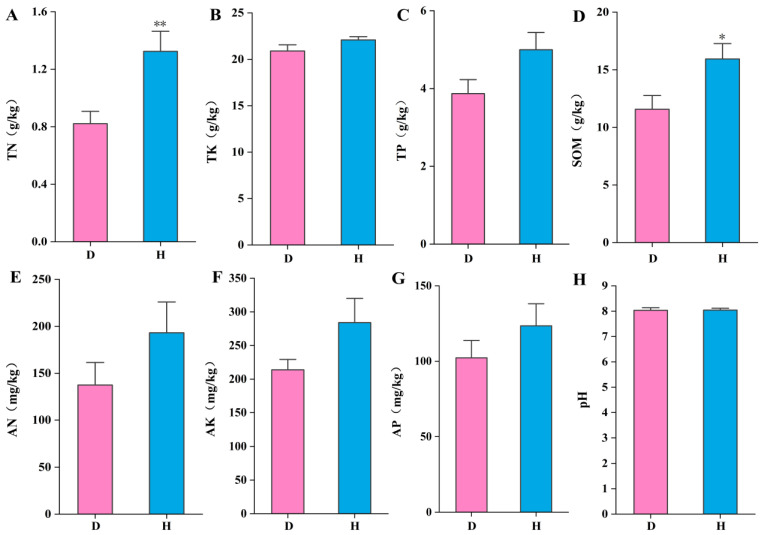
The analysis of soil physicochemical properties, where (**A**) is total nitrogen (TN), (**B**) is total potassium (TK), (**C**) is total phosphorus (TP), (**D**) is soil organic matter (SOM), (**E**) is alkali-hydrolyzable nitrogen (AN), (**F**) is available phosphorus (AP), (**G**) is available potassium (AK), and (**H**) is pH values. Letters indicate significant differences among different soil samples by ANOVA at *p* * < 0.05, *p* ** < 0.01 vs. H group (n = 17 in each group). H is healthy samples; D is diseased samples.

An exhaustive evaluation of the soil’s nutrient profile was undertaken, focusing on the primary chemical properties, as depicted in Figure 2. These encompassed TN, TK, TP, SOM, AN, AP, AK, and pH values. Statistical analysis revealed negligible differences in the rhizosphere soil characteristics between H and D, save for TN and SOM. Notably, the mean concentrations of TN (Figure 2A) and SOM (Figure 2D) were substantially higher in the H when contrasted with those from D.

### 3.2. The Number of OTUs and Alpha Diversity of Fungi

**Figure 3:**
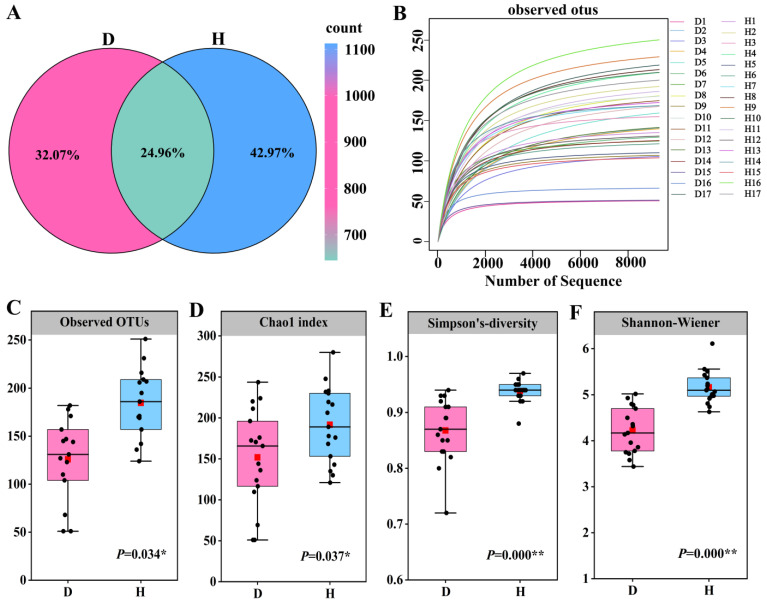
(**A**) The OTU distribution Venn diagram; (**B**) The saturation of rarefaction curves; (**C**) The Observed Operational Taxonomic Units (OTUs); (**D**) The Chao1 richness index; (**E**) Simpson’s diversity index; (**F**) The Shannon-Wiener diversity index. Utilizing Illumina sequencing technology, Venn diagram is at a 97% sequence similarity threshold, and the OTU distribution Venn diagram shows that the D group harbored 830 unique OTUs, accounting for 32.07% of the total, while the H group had 1112 unique OTUs, comprising 42.97% of the total. Only 646 OTUs were shared between the two groups, representing 24.96% of the total. These data clearly indicate a higher fungal richness in the H group compared to the D group. The rarity curve saturation in (**B**) suggests that the sequencing depth was sufficient to capture the full spectrum of fungal community diversity in the rhizospheric soil. Consequently, these findings are deemed to accurately represent the actual state of the fungal communities and are suitable for further analysis. (**C**–**F**) illustrate the alpha diversity of the soil fungal communities within each sample, evaluated by multiple metrics, including the number of observed OTUs, the Chao1 richness index, the Simpson diversity index, and the Shannon-Wiener diversity index. Notably, significant differences were found between the D and H groups for all indices, with the H soils exhibiting a higher level of diversity than the D soils. * indicate significant differences among different soil samples by ANOVA at *p* < 0.05, ** indicate significant differences among different soil samples by ANOVA at *p* < 0.01.

Fungal OTUs and alpha diversity derived from Illumina sequencing technology, a total of 2,966,289 high-quality fungal tags were procured from the 34 rhizosphere soil samples under study, with an average of 83,042 and 90,978 tags in the D and H groups, respectively. The collective number of operational taxonomic units (OTUs) at a 97% sequence similarity threshold amounted to 2588 across all samples. As illustrated by the OTU distribution Venn diagram (Figure 3A), the D group harbored 830 exclusive OTUs, which constituted 32.07% of the overall count, while the H group was found to contain 1112 unique OTUs, comprising 42.97% of the total. A mere 646 OTUs were common to both the D and H groups, making up only 24.96% of the total 2588 OTUs identified within the sampled regions. The data clearly suggest a higher fungal richness in the H group compared to the D group. The saturation of rarefaction curves (Figure 3B) indicates that the sequencing depth was adequate to capture the full extent of fungal community diversity in the rhizosphere soils. Therefore, these findings are considered to accurately represent the fungal community’s actual state and are suitable for further analytical pursuits.

The alpha diversity of the soil fungal communities within each sample was evaluated via several metrics, including the observed operational taxonomic units (OTUs) (Figure 3C), the Chao1 richness index (Figure 3D), Simpson’s diversity index (Figure 3E), and the Shannon-Wiener diversity index (Figure 3F). Notably, there were pronounced differences in all indices between the D and H groups, with the H soils demonstrating a higher level of diversity compared to the D soils.

### 3.3. Community Composition and Relative Abundance of Fungi

**Figure 4:**
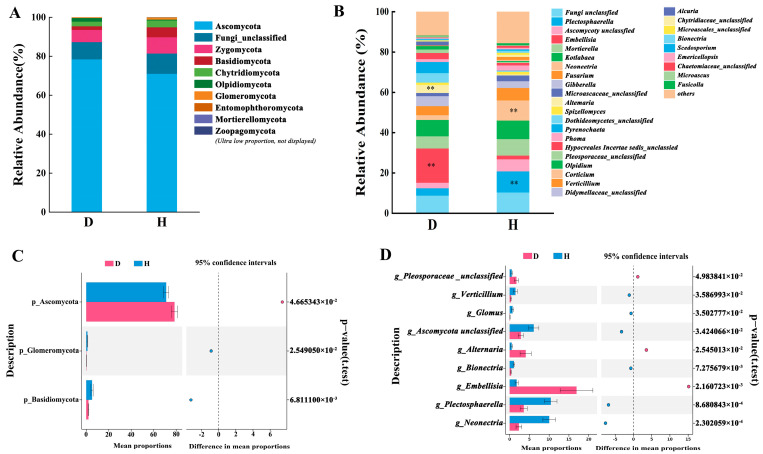
(**A**,**B**): Variation in microbial abundance at the phylum and genus levels; (**C**,**D**): H and D microbes with differential abundance at the phylum and genus levels. (**A**–**D**) collectively illustrate the composition and relative abundance of the fungal community in the rhizospheric soil of wolfberry. (**A**) reveals the distribution of over nine distinct fungal phyla, with Ascomycota being the predominant group, averaging a 74.78% abundance, followed by Zygomycota, Basidiomycota, and Chytridiomycota. (**C**) discloses significant differences in the fungal communities between healthy (H) and root-rot (D) samples, where the abundance of Ascomycota increased by 10.43% in the D samples, while the abundance of Basidiomycota and Glomeromycota significantly decreased. (**B**,**D**) further analyze the distribution of fungal genera, with 332 genera exhibiting significant changes under different treatments; a higher abundance of *Plectosphaerella*, *Mortierella*, *Kotlabaea*, and *Neonectria* was observed in healthy samples, whereas the abundance of *Embellisia* and *Alternaria* significantly increased in D samples. These findings indicate that root rot significantly alters the structure of the fungal community in the rhizospheric soil, exerting a substantial impact on agricultural production. ** indicate significant differences among different soil samples by ANOVA at *p* < 0.05.

The distribution of fungal phyla within the rhizosphere soil across the various treatments exhibited notable variability, encompassing more than nine distinct phyla. Predominant among these were Ascomycota, Zygomycota, Basidiomycota, Chytridiomycota, Olpidiomycota, Glomeromycota, Entomophthoromycota, Mortierellomycota, and Zoopagomycota, as illustrated in Figure 4A. Across all samples, Ascomycota emerged as the predominantly represented phylum, averaging an abundance of 74.78%. This was succeeded by Zygomycota, Basidiomycota, and Chytridiomycota, with average abundances of 7.27%, 3.56%, and 2.92%, respectively. Collectively, these four phyla constituted over 88% of the fungal assemblage observed across the different treatments. A comparative analysis of the fungal community abundance at the phylum level between the D and H samples (*p* < 0.05) indicated a significant elevation in the prevalence of Ascomycota in the D group, showing a 10.43% increase in abundance in the rot root-infected soil, reaching a level of 78.48%. Conversely, a marked reduction in the abundance of Basidiomycota and Glomeromycota was observed in the D group compared to the H group, with declines of 62.28% and 79.09%, respectively (Figure 4C). Moreover, approximately 9.65% of the fungi remained unclassified.

At the genus level, a total of 332 genera were distinguished within the 34 rhizosphere soil samples, manifesting substantial variations in the fungal communities associated with *L. barbarum* under different treatments. Predominant genera included *Embellisia* (9.34%), *Kotlabaea* (8.69%), *Mortierella* (7.18%), *Plectosphaerella* (6.99%), *Neoelectronics* (6.17%), and *Fusarium* (5.31%), each with relative abundances exceeding 5% (refer to Figure 4B). It is noteworthy that an appreciable proportion (16.36%) of the community remained unidentified. In the healthy samples (H), the dominant genera were *Plectosphaerella* (10.41%), *Mortierella* (8.25%), *Kotlabaea* (9.22%), and *Neonectria* (10.00%), displaying increased average relative abundances. By contrast, the D samples were predominantly characterized by *Embellisia* (16.92%), *Kotlabaea* (8.16%), and Mortierella (6.10%). Statistical significance analysis (*p* < 0.05) indicated that the average relative abundances of *Plectosphaerella*, *Neoelectronics*, *Bioelectronics*, *Glomus*, and *Verticillium* were substantially higher in the healthy samples as compared to the diseased ones. When contrasting D with H, the relative abundances of *Embellisia* and *Alternaria* demonstrated remarkable increases of 89.59% and 87.41%, respectively (as illustrated in Figure 4D).

### 3.4. NMDS and LEfSe of Fungal Community Composition

**Figure 5:**
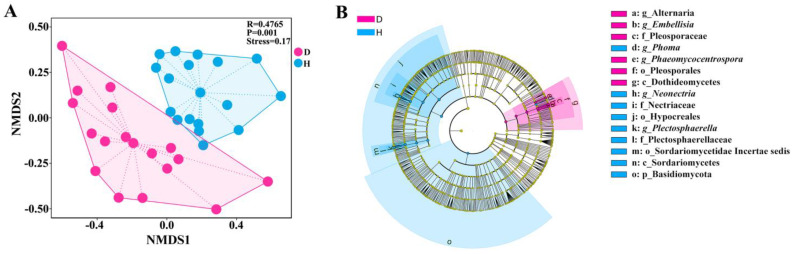
Relative abundances of fungal classes in rhizosphere soil samples. NMDS analysis (**A**), coupled with LEfSe analysis (**B**), has elucidated significant distinctions in the fungal communities of the rhizosphere soil between healthy (H) and root-rot infected (D) in *L. barbarum* plants. The NMDS analysis revealed a notable differentiation in the fungal community structure between the two groups, with a stress value of 0.17, indicating statistically significant results. The fungal communities in the healthy plants exhibited greater consistency, whereas those in the infected plants were more dispersed. The LEfSe analysis further revealed significant differences in the relative abundance and distribution of fungi at the phylum, class, family, and genus levels between the H and D soil samples. Notably, the H samples registered higher LDA scores for Basidiomycota, Glomeromycota, Eurotiomycetes, Sordariomycetes, Chytridiomycetes, and Glomeromycetes, whereas the D samples were enriched with Dothideomycetes, Cucurbitariaceae, and Pleosporaceae. At the genus level, the H samples were characterized by the highest LDA scores for *Phoma*, *Bionectria*, *Neonectria*, *Plectosphaerella*, *Verticillium*, and *Glomus*, while the D samples were distinguished by the prominence of *Pyrenochaeta*, *Didymella*, *Alternaria*, *Embellisia*, and *Phaeomycocentrospora*.

Non-metric Multidimensional Scaling (NMDS) was employed to analyze the fungal communities present in the rhizospheric soils of both healthy and diseased *L. barbarum* based on the feature set employed. The stress value functions as a metric for assessing the reliability of the NMDS outcomes. As depicted in Figure 5A, the stress value for the NMDS analysis of the D and H fungal communities was 0.17, which is below the conventional threshold of 0.2. This indicates that the results are statistically significant and possess considerable explanatory capability. The analysis revealed a notable differentiation in the fungal community structures between the D and H groups, with an R-value of 0.4765 and a *p*-value below the 0.01 threshold. It is worth mentioning that there were overlapping zones between these two groups, suggesting certain commonalities in the fungal community profiles across individual samples. The fungal community structure within the rhizosphere of healthy plants seemed to be more consistent, with the samples in the H group displaying a tighter clustering. Conversely, the samples in the D group demonstrated a higher degree of dispersion.

Linear Discriminant Analysis (LDA) facilitated by LEfSe, with a threshold of LDA score greater than 3.5, was employed to discern fungal biomarker microbiomes across various taxonomic levels, from phylum to genus, between the soils of rot-rooted and healthy roots of *L. barbarum*. As depicted in Figure 5B, the LDA scores revealed significant differentiation in the phylum-level representation of Basidiomycota and Glomeromycota between the H and D soil samples. At the class level, H soil samples exhibited notably higher LDA scores for Eurotiomycetes, Sordariomycetes, Chytridiomycetes, and Glomeromycetes, in contrast to the increased prevalence of Dothideomycetes in the D soil samples. In terms of family distribution, the relative abundances of Bionectriaceae, Nectriaceae, Plectosphaerellaceae, Spizellomycetaceae, and Glomeraceae were significantly greater in the H soil as compared to the D soil, while Cucurbitariaceae and Pleosporaceae were more predominant in the latter. At the genus level, the H soil microbiomes were characterized by higher LDA scores for *Phoma*, *Bionectria*, *Neonectria*, *Plectosphaerella*, *Verticillium*, and *Glomus*, whereas the D soil was marked by the predominance of *Pyrenochaeta*, *Didymella*, *Alternaria*, *Embellisia*, and *Phaeomycocentrospora*.

### 3.5. Effects of Soil Chemical Properties on Fungal Community Structure

**Figure 6:**
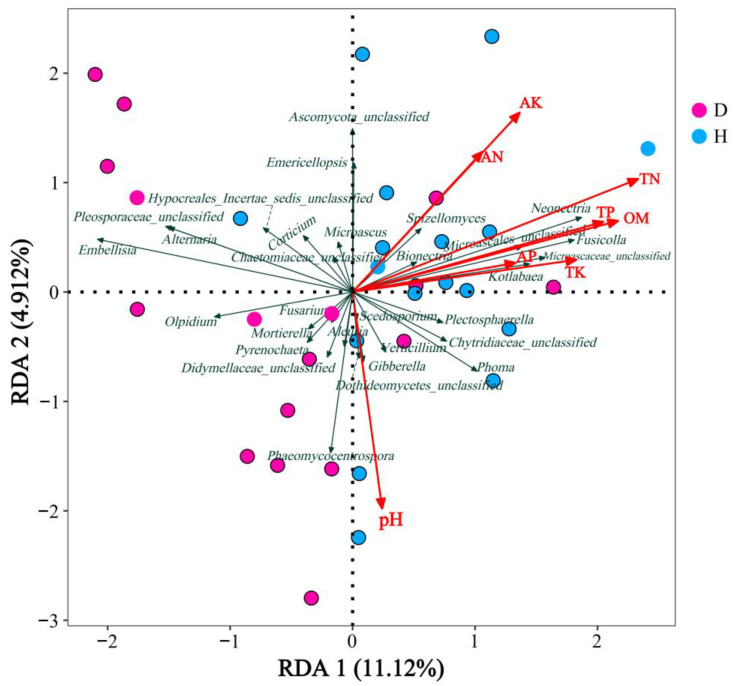
RDA of chemical properties and fungal communities in rhizosphere soils. RDA collectively elucidates the influence of soil chemical properties on the fungal community structure in the rhizosphere soil of *L. barbarum*. The RDA revealed that chemical attributes such as total nitrogen (TN), soil organic matter (SOM), and total phosphorus (TP) significantly affect the relative abundance of fungal genera, with TN emerging as a predominant influencing factor, positively correlating with the microbial communities associated with healthy plants. Conversely, pH exerted a pivotal impact on the distribution of fungi in the rhizosphere of diseased plants.

Redundancy Analysis (RDA) was conducted to delineate the interplay between the chemical attributes and fungal community composition within the rhizosphere soils (Figure 6). The analysis elucidated that soil chemical properties exerted notable influences on the genus-level relative abundance of fungal communities. As indicated by RDA, the variance in microbial assemblages explained by the primary axis was 11.12%, while the secondary axis accounted for 4.91% of the variation. The findings highlighted that Total Nitrogen (TN) was the predominant factor affecting the relative abundance of the 30 predominant genera, succeeded by Soil Organic Matter (SOM), Total Phosphorus (TP), Available Potassium (AK), pH, and Total Potassium (TK). Predominantly, TN, SOM, TP, AK, and TK were positively correlated with microbial communities associated with robust plant health. Conversely, pH emerged as a pivotal determinant influencing the distribution of fungal communities in the rhizosphere of plants under disease stress. The elements TN, SOM, TP, TK, AK, Available Phosphorus (AP), and Ammonium Nitrogen (AN) were found to be positively associated with genera such as *Plectosphaerella*, *Neonectria*, *Fusicolla*, *Kotlabaea*, and *Spizellomyces*, while displaying positive correlations with *Phaeomycocentrospora*, *Alternaria*, *Embellisia*, and *Fusarium*. Notably, the relative abundances of *Phaeomycocentrospora*, *Gibberella*, *Mortierella*, and *Fusarium* were enhanced in conjunction with increased pH values, whereas the relative abundances of *Neonectria*, *Fusicolla*, *Kotlabaea*, and *Spizellomyces* were inversely associated with pH levels.

Subsequent Variation Partitioning Analysis (VPA) was utilized to unravel the impact of chemical characteristics on the microbial community structure within the rhizosphere soils of *L. barbarum* (Figure S1). According to the VPA, approximately 25% of the community variation could be ascribed to chemical properties, with pH + SOM, phosphorus, and nitrogen contributing to 5%, 4%, and 3% of the variance, respectively. Moreover, pH and organic matter were identified as the most influential factors shaping the composition of the microbial community.

### 3.6. Functions of Soil Fungi Associated with the Changes in Community Structure

The functional roles of soil fungi in relation to modifications of community structures were evaluated through FUNGuild analysis, which adeptly anticipated the functions of these fungi in both the healthy and root-rotten *L. barbarum* plants. Drawing from database annotations, FUNGuild categorizes fungi into eight distinct trophic groups in this study: Pathotroph (P), Saprotroph (Sa), Symbiotroph (Sy), Pathotroph-Saprotroph-Symbiotroph (P-Sa-Sy), Saprotroph-Symbiotroph (Sa-Sy), Pathotroph-Saprotroph (P-Sa), Pathotroph-Symbiotroph (P-Sy), and unknown. These categories are generally manifested as three independent trophic modes and complex multitrophic modes involving two or more trophic interactions, which can be further divided into 68 subguilds.

**Figure 7:**
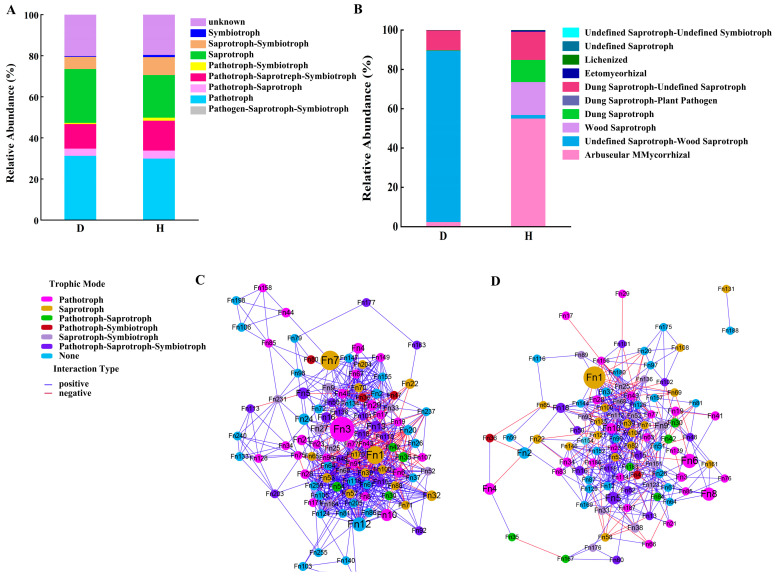
The predicted trophic mode and subguilds bar graph by FUNguild (**A**,**B**) and Network diagram of fungal taxonomic trophic model interactions by cytoscape (**C**,**D**). In (**A**,**B**), we have evaluated the functional roles of soil fungi in both healthy and root-rot infected *L. barbarum* plants, categorizing the fungi into eight trophic modes and sixty-eight subgroups. C and D are Network diagrams drawn according to trophic mode in the H and D groups, where node represents trophic mode classification, node size represents OTU data values, Fn 1, Fn 2, Fn 3, etc. are abbreviations for OTU, blue lines represent two OTU with positive relationships, and red lines represent negative relationships between two OTU, through the analysis conducted via FUNGuild. The study revealed that 80.4% of the nutritional modes were understood in the healthy plants, while 79.9% were predicted in the infected ones. Notably, the pathotrophic (P) and saprotrophic (Sa) nutritional types exhibited significant positive and negative interactions, respectively, in both the healthy (H) and infected (D) plants. Pathogens were more prevalent in the infected plants, whereas the healthy plants predominantly harbored mutualistic and saprotrophic fungi.

As predicted by FUNGuild, a comprehensive understanding was gained of 80.4% of the trophic modes present in healthy plants. A total of 242 genera were found to be enriched, with the independent modes P and Sa dominating the distribution at 29.8% and 20.7%, respectively, followed by the combined P-Sa-Sy mode at 14.5%. The trophic modes Sa-Sy, P-Sa, P-Sy, and Sy were predicted to account for 8.8%, 3.9%, 1.5%, and 1.0% of the fungi, respectively. In the infected specimens, where 242 genera were similarly enriched, FUNGuild predicted trophic modes for 79.9% of the fungi. Here, the fully integrated P-Sa-Sy mode comprised 11.9%, while the independent modes P and Sa were again predominant, accounting for 31.2% and 26.1%, respectively. Notably, the H treatment witnessed a reduction in the prevalence of the independent trophic modes P and Sa, concomitant with an increase in the representation of one independent mode (Sy) and four multitrophic modes (P-Sa-Sy, Sa-Sy, P-Sa, and P-Sy) among the soil fungi (Figure 7A).

The prevalence of plant pathogens within subguilds was predominantly high, averaging 27.0% in both the healthy and infected plants, with the incidence of pathogens being notably higher in the latter. Among the healthy plants, prominent subgroups comprised Endophyte-Dung Saprotroph-Lichen Parasite-Litter Saprotroph-Plant Pathogen-Soil Saprotroph-Wood Saprotroph, along with Arbuscular Mycorrhizal fungi. In contrast, the infected plants exhibited a higher relative abundance of subclasses such as Animal Parasite-Fungal Parasite, Dung Saprotroph-undefined saprophyte, and Endophyte-Lichen Parasite-Plant Pathogen-Undefined saprophyte. Notably, *Embellisia*, a subclass of Plant Pathogen (P), represented the most abundant group in the infected wolfberry plants, accounting for 15.6% of the relative abundance, followed by *Pyrenochaeta* at 8.7% and *Kotlabaea* at 8.0%. *Kotlabaea*, classified under the Undefined *Saprotroph* subclass of Sa, was the predominant species in healthy plants, with a relative abundance of 10.7% (Figure 7B). Meanwhile, an intricate web of taxonomic interactions was observed, characterized by a complex array of direct and indirect positive and negative associations among fungal taxa across various trophic modes (Figure 7C,D).

## 4. Discussion

Microorganisms are pivotal in the cycling of soil nutrients, serving as both a repository and a dynamic pool for these nutrients. Numerous factors influence soil. As one of the significant plants for continuous cultivation and a valued medicinal herb, *L. barbarum* has seen extensive fertilizer application by growers to enhance its yield and quality. robiota, including plant genotypes, soil types, and fertilization practices [36]. Such fertilization can alter the physical and chemical properties of the soil, prompting microorganisms to evolve a myriad of strategies to adapt to diverse soil environments [37]. Fungi, in particular, are known for their role in decomposing plant residues and influencing plant disease occurrence. There is a well-established relationship between the health of plant roots and the fungal community structure. This study, therefore, aims to elucidate the changes in the fungal community structure in the rhizosphere soil of healthy *L. barbarum* plants in the Qaidam Basin under cultivation conditions after root rot infection, as well as the interplay with soil physicochemical properties. The findings could provide a theoretical basis for microbial control of soil-borne diseases.

Research has confirmed that a higher microbial community diversity index signifies a more complex community structure and greater systemic stability, enhancing the capacity to withstand environmental changes [38]. This paper evaluates the alpha diversity of the fungal communities in the rhizosphere soil of healthy and diseased *L. barbarum* plants through observed OTUs, the Chao1 index, Simpson’s diversity, and the Shannon-Wiener index, with the H soil demonstrating superior diversity. A prior study noted that the diversity and richness of the fungal community in the rhizosphere soil of healthy potato plants were significantly greater than those in diseased plants, creating a more conducive external environment for potato resistance to *R. solani* [39]. Furthermore, it has been observed that microbial diversity in soil affected by banana *Fusarium* wilt is reduced compared to healthy soil. These observations may be attributed to changes in the rhizosphere soil environment following the onset of plant disease, disturbing the equilibrium between rhizosphere fungi and roots and affecting overall fungal diversity [40]. Zhang et al. have demonstrated that a diverse assemblage of rhizosphere soil fungi is more likely to contribute to the early stages of wheat root rot [41]. Consequently, the severity of root rot in plants is escalating, and it is proposed that soil-borne diseases can decrease soil microbial diversity. Studies have confirmed that different microorganisms play significant roles in agricultural production. When soil-borne diseases occur, the microbial diversity and the dynamic balance among communities in the rhizosphere soil of healthy and diseased plants often exhibit substantial differences [42]. Based on NMDS and LEfSe analysis, this study reveals significant variations in the fungal community compositions between healthy and afflicted samples collected from identical locations. Notably, there were overlapping zones between these two groups, suggesting certain commonalities in the fungal community profiles across individual samples. The fungal community structure within the rhizosphere of robust plants demonstrated high aggregation. Conversely, the samples in the D group displayed a higher degree of dispersion, which indicated that the microbial structure of H rhizosphere soil was very stable. This phenomenon can be ascribed to the understanding that root rot is not exclusively caused by a solitary pathogen. Instead, it is likely the outcome of a complex interplay among various pathogens and the host plant. The existence of specific individual variations within the diseased group may introduce nuanced alterations in the pathogen interaction dynamics, thereby accounting for the observed degree of dispersion [43].

Analysis of the soil fungal community composition distribution in the samples indicates a notable alteration in the fungal assemblage within the rhizosphere soil of *L. barbarum* plants affected by disease. At the phylum level, Ascomycota was the predominant group in both the healthy (H) and diseased (D) cohorts, with a significantly increased prevalence observed in the latter. Xie Yuqing et al. [44] employed high-throughput sequencing to examine the changes in the fungal community structure of garlic rhizosphere soil during the onset of root rot disease in Jimusaer County, Xinjiang, concluding that the high abundance of Ascomycota was closely associated with garlic root decay. Similarly, Song Xuhong et al. [45] utilized Illumina high-throughput sequencing technology to analyze the fungal community composition in the rhizosphere soil of Coptis chinensis affected by root rot, revealing a significantly higher relative abundance of Ascomycota in the rhizosphere soil of diseased plants compared to healthy ones, while the relative abundance of Zygomycota was significantly reduced. Chen Haisheng’s [46] findings indicated that after infection with root rot, the relative abundance of Ascomycota in the rhizosphere soil of kiwi plants increased by 115.71% compared to non-infected plants, while the relative abundance of Basidiomycota decreased by 94.66%. These results are consistent with the aforementioned studies, suggesting that the root exudates of healthy *L. barbarum* plants can inhibit the proliferation of Ascomycota and promote the growth of Basidiomycota. However, this balance between the plant roots and the rhizosphere soil microbiota is disrupted upon infection with root rot, thereby fostering the proliferation of Ascomycota in the rhizosphere soil [47].

At the genus level, the predominant genera in the healthy samples (H) were *Plectosphaerella* (10.41%), *Mortierella* (8.25%), *Kotlabaea* (9.22%), and *Neonectria* (10.00%), exhibiting enhanced average relative abundances. In stark contrast, the D samples were predominantly defined by *Embellisia* (16.92%), *Kotlabaea* (8.16%), and *Mortierella* (6.10%). *Plectosphaerella* has been identified as a primary factor contributing to the continuous cropping barrier of plants such as peach trees, being significantly more prevalent in the H group than in the D group. Concurrently, it was observed that the H group also harbored a higher abundance of beneficial fungi, including the genus *Mortierella* and *Glomus* [48]. In contrast, the D group exhibited a predominant presence of the genus *Embellisia*, which has been reported as a major pathogen causing yellow dwarf root rot in *Astragalus adsurgens* [49] and root rot in *Allium sativum* [50]. Additionally, *Alternaria* experienced a marked increase of 87.41% in the D group, a genus commonly found as both a soil saprophyte and a plant pathogen. Therefore, root rot infections in *L. barbarum* were found to be associated with an elevated incidence of pathogenic fungi, whereas the presence of beneficial and symbiotic fungi was more pronounced in the healthier counterparts.

The composition of the fungal community corresponds accurately to the predictions of FUNguild, which revealed that the H treatment led to a diminished prevalence of the independent trophic modes Pathotroph (P) and Saprotroph (Sa), accompanied by an enhanced representation of the Symbiotroph (Sy) mode and four multitrophic modes (P-Sa-Sy, Sa-Sy, P-Sa, and P-Sy) among the soil fungi. It has been proposed in research that the enrichment of pathogenic bacteria is but the initial step towards plant disease manifestation. It is only when such enrichment reaches a certain threshold, leading to an imbalance in the microbial community structure and a decrease in diversity levels unable to counteract the destructive force of the pathogens, that the onset of disease in plants becomes ultimate [51]. In the H treatment group, although there was an enrichment of pathogenic bacteria, it is plausible that the relative increase in the abundance of transitional fungal groups, such as the soil saprotrophic-symbiotic, pathogen-saprotrophic-symbiotic, and pathogenic-saprotrophic-symbiotic trophic modes, may have prevented the pathogens from multiplying to a disease-causing magnitude [52,53].

Moreover, it has been documented that the fungal assemblage of a given tree species tends to maintain a degree of constancy across certain geographical extents despite being sourced from varied sampling localities [54]. In the study, the mean concentrations of TN (Figure 2A) and SOM (Figure 2D) were substantially higher in the healthy soil samples when contrasted with those from the diseased counterparts. Redundancy Analysis (RDA) outcomes revealed correlations between the abundances of certain genera and soil characteristics such as pH, TN, SOM, TP, AK, and TK. Predominantly, TN, SOM, TP, AK, and TK were positively correlated with microbial communities associated with robust plant health. Conversely, pH emerged as a pivotal determinant influencing the distribution of fungal communities in the rhizosphere of plants under disease stress.

## 5. Conclusions

In summary, our findings categorically delineate that the fungal community structure in the rhizosphere soil of *L. barbarum* in the Qaidam Basin undergoes pronounced alterations subsequent to the onset of root rot disease. The diversity of fungal communities in the rhizosphere soil of affected plants is notably lower compared to that of healthy plants. The fungal genera most significantly impacting both healthy and affected plants are Embellisia, Alternaria, Plectosphaerella, and Mortierella. Concurrently, there is a marked increase in the Pathotroph (P) and Saprotroph (Sa) categories in the D group relative to the H group, whereas the independent Symbiotroph (Sy) and the four multitrophic modes (P-Sa-Sy, Sa-Sy, P-Sa, and P-Sy) exhibit a decline. The enrichment of pathogenic trophic fungi in the rhizosphere disrupts the soil microbiota balance, serving as a primary catalyst for the onset of root rot in *L. barbarum*. This study contributes to a deeper understanding of the microecological mechanisms underpinning the occurrence of root rot, providing a theoretical foundation for disease prevention and control. Nevertheless, the journey towards the effective biological control of root rot and other soil-borne diseases remains arduous. The alterations in microbial populations are a dynamic process, necessitating continuous monitoring and forecasting at both depth and breadth to promptly detect changes in the rhizosphere soil trophic types. Utilizing beneficial microbial agents to enhance the soil microecological environment and to fully capitalize on the biocontrol potential of strains can control pathogen proliferation.

## Figures and Tables

**Table 1:** Details of the nine sampling collecting sites.

Location	Abbreviation	Latitude	Longitude	Altitude (m)	Sample Size (Number)
Nuomuhong farm, Zongjia town, Dulan county	XY	36.39127	96.458381	2768.33	12
	NY	36.436204	96.484412	2752.84	12
	NSI	36.440156	96.313511	2718.31	12
	NW	36.4289	96.267501	2719.58	12
	NS	36.440189	96.354075	2736.39	12
	NE	36.448763	96.475582	2718.97	6
Dagele township, Golmud city	KP	36.437893	95.717555	2733.58	12
Guolemude town, Golmud city	GEM	36.417216	94.304577	2767.54	12
Huaitoutala town, Delingha city	WSJ	37.338021	96.695822	2812.01	12
Total					102

## Data Availability

The original contributions presented in this study are included in the article. Further inquiries can be directed to the corresponding author.

## Supplementary Materials

The following supporting information can be downloaded at: https://www.mdpi.com/article/10.3390/microorganisms12122447/s1, Figure S1: Subsequent Variation Partitioning Analysis (VPA) was utilized to unravel the impact of chemical characteristics on the microbial community structure within the rhizosphere soils of *L. barbarum* (Figure S1). According to the VPA, approximately 25% of the community variation could be ascribed to chemical properties, with pH + SOM, phosphorus, and nitrogen contributing to 5%, 4%, and 3% of the variance, respectively. Moreover, pH and organic matter were identified as the most influential factors shaping the composition of the microbial community.

## Abbreviations

D, Diseased plants (from *L. barbarum*); H, Healthy plants (from *L. barbarum*); OTU, Operational Taxonomic Unit; NMDS, Non-metric Multidimensional Scaling; LEfSe, Linear Discriminant Analysis Effect Size; RDA, Redundancy Analysis; VPA, Variation Partitioning Analysis; P, Pathotroph (in FUNGuild analysis); Sa, Saprotroph (in FUN Guild analysis); Sy, Symbiotroph (in FUN Guild analysis); TN, total nitrogen; TP, total phosphorus; TK, total potassium; AN, alkali-hydrolyzable nitrogen; AK, available potassium; AP, Available Phosphorus; SOM, soil organic matter; PCR, Polymerase Chain Reaction; ITS2, Internal Transcribed Spacer 2; PEAR, Paired-End reAd merger software; QIIME2, Quantitative Insights Into Microbial Ecology 2; SPSS, Statistical Package for the Social Sciences.

## References

[1] Skenderidis P., Mitsagga C., Giavasis I., Petrotos K., Lampakis D., Leontopoulos S. (2019). The in vitro antimicrobial activity assessment of ultrasound assisted *Lycium barbarum* fruit extracts and pomegranate fruit peels. J. Food Meas. Charact..

[2] Cho H., Lee D.H., Jeong D., Jang J., Son Y., Lee S., Kim H. (2024). Study on Betaine and Growth Characteristics of *Lycium chinense* Mill. in Different Cultivation Environments in South Korea. Plants.

[3] Jiang C., Chen Z., Liao W., Zhang R., Chen G., Ma L., Yu H. (2024). The Medicinal Species of the *Lycium* Genus (*Goji berries*) in East Asia: A Review of Its Effect on Cell Signal Transduction Pathways. Plants.

[4] Jia C., An Y., Du Z., Gao H., Su J., Xu C. (2023). Differences in Soil Microbial Communities between Healthy and Diseased *Lycium barbarum* cv. Ningqi-5 Plants with Root Rot. Microorganisms.

[5] Zhang Z., He K., Zhang T., Tang D., Li R., Jia S. (2019). Physiological responses of Goji berry (*Lycium barbarum* L.) to saline-alkaline soil from Qinghai region, China. Sci. Rep..

[6] Amagase H., Farnsworth N.R. (2011). A review of botanical characteristics, phytochemistry, clinical relevance in efficacy and safety of *Lycium barbarum* fruit (goji). Food Res. Int..

[7] Feng Z., Xiao Y., Li N., Gao Q., Wang J., Chen S., Xing R. (2023). Effects of root rot on microbial communities associated with goji berry (*Lycium barbarum*) in the Qaidam Basin, China. Eur. J. Plant Pathol..

[8] Uwaremwe C., Yue L., Liu Y., Liu Y., Tian Y., Zhao X., Wang Y., Xie Z., Zhang Y., Cui Z. (2020). Molecular identification and pathogenicity of Fusarium and Alternaria species associated with root rot disease of wolfberry in Gansu and Ningxia provinces, China. Plant Pathol..

[9] Wang Q., Wang J., Zhang Z., Li M., Wang D., Zhang P., Na L., Yin H. (2024). Microbial metabolic traits drive the differential contribution of microbial necromass to soil organic carbon between the rhizosphere of absorptive roots and transport roots. Soil Biol. Biochem..

[10] Zhang J.H., Zheng G. (2016). Soil nematode community structure in the rhizosphere of *Lycium barbarum*. J. Appl. Ecol..

[11] Rashida M., Mujawara L., Shahzade T., Almeelbi T., Iqbal M., Oves M. (2016). Bacteria and fungi can contribute to nutrients bioavailability and aggregate formation in degraded soils. Microbiol. Res..

[12] Taheri A., Hamel C., Gan Y. (2015). Pyrosequencing reveals the impact of foliar fungicide application to chickpea on root fungal communities of durum wheat in subsequent year. Fungal Ecol..

[13] Monkai J., Purahong W., Nawaz A., Wubet T., Hyde K., Goldberg S., Mortimer P., Xu J., Harrison R. (2022). Conversion of rainforest to rubber plantations impacts the rhizosphere soil mycobiome and alters soil biological activity. Land. Degrad. Dev..

[14] Zhao L., Li H., Liu Z., Hu L., Xu D., Zhu X., Mo H. (2024). Quality Changes and Fungal Microbiota Dynamics in Stored Jujube Fruits: Insights from High-Throughput Sequencing for Food Preservation. Foods.

[15] Wang M., Sun H., Dai H., Xu Z. (2024). Characterization of Plant-Growth-Promoting Rhizobacteria for Tea Plant (*Camellia sinensis*) Development and Soil Nutrient Enrichment. Plants.

[16] Li X., Li J., Qi Y., Guo W., Li X., Li M. (2017). Effects of naked barley root rot on rhizosphere soil microorganisms and enzyme activity. Acta Ecol. Sin..

[17] Santoyo G. (2022). How plants recruit their microbiome? New insights into beneficial interactions. J. Adv. Res..

[18] Zhao Y., Chen H., Sun H., Yang F. (2024). In the Qaidam Basin, Soil Nutrients Directly or Indirectly Affect Desert Ecosystem Stability under Drought Stress through Plant Nutrients. Plants.

[19] Liu S., Zheng J. (2024). Adaptive strategies based on shrub leaf-stem anatomy and their environmental interpretations in the eastern Qaidam Basin. BMC Plant Biol..

[20] Qiu L., Zhang X., Cheng J., Yin X. (2010). Effects of black locust (*Robinia pseudoacacia*) onsoil properties in the loessial gully region of the loess plateau, China. Plant Soil..

[21] Li J., Zheng Q., Liu J., Pei S., Yang Z., Chen R., Ma L., Niu J., Tian T. (2024). Bacterial–fungal interactions and response to heavy metal contamination of soil in agricultural areas. Front. Microbiol..

[22] Xu F., Chu C., Xu Z. (2020). Effects of different fertilizer formulas on the growth of loquat rootstocks and stem lignification. Sci. Rep..

[23] Li Y., Fang F., Wei J., Wu X., Cui R., Li G., Zheng F., Tan D. (2019). Humic acid fertilizer improved soil properties and soil microbial diversity of continuous cropping peanut: A three-year experiment. Sci. Rep..

[24] Yang M., Ji S., Duan G., Fan G., Li J., Wang Z. (2024). Staining methods on arbuscular mycorrhizal fungi in *Lycium barbarum* roots and the relationship between colonization rate and soil factors. J. J. Shandong Univ. (Nat. Sci.).

[25] Pei G., Zhu Y., Wen J., Pei Y., Li H. (2019). Vinegar residue supported nanoscale zero-valent iron: Remediation of hexavalent chromium in soil. Environ. Pollut..

[26] Karlsson I., Friberg H., Steinberg C., Persson P. (2014). Fungicide effects on fungal community composition in the wheat phyllosphere. PLoS ONE..

[27] Wang X., Wang Z., Jiang P., He Y., Mu Y., Lv X., Zhuang L. (2018). Bacterial diversity and community structure in the rhizosphere of four *Ferula* species. Sci. Rep..

[28] Martin M. (2011). Cutadapt removes adapter sequences from high-throughput sequencing reads. EMBnet J..

[29] Chaudhary P., Bhattacharjee A., Khatri S., Dalal R., Kopittke P., Sharma S. (2024). Delineating the soil physicochemical and microbiological factors conferring disease suppression in organic farms. Microbiol. Res..

[30] Callahan B., McMurdie P., Rosen M., Han A., Johnson A., Holmes S.P. (2016). DADA2: High-resolution sample inference from Illumina amplicon data. Nat. Methods.

[31] Bolyen E., Rideout J., Dillon M. (2019). Reproducible, interactive, scalable and extensible microbiome data science using QIIME 2. Nat. Biotechnol..

[32] Nguyen N., Song Z., Bates S., Branco S., Tedersoo L., Menke J., Schilling J., Kennedy P. (2016). FUNGuild: An open annotation tool for parsing fungal community datasets by ecological guild. Fungal Ecol..

[33] Segata N., Izard J., Waldron L., Gevers D., Miropolsky L., Garrett W. (2011). Metagenomic biomarker discovery and explanation. Genome Biol..

[34] de Vries F., Griffiths R., Bailey M. (2018). Soil bacterial networks are less stable under drought than fungal networks. Nat. Commun..

[35] Amand J., Fehlmann T., Backes C., Keller A. (2019). DynaVenn: Web-based computation of the most significant overlap between ordered sets. BMC Bioinform..

[36] Liu H., Xiong W., Zhang R., Huan X., Wang D., Shen Q. (2018). Continuous application of different organic additives can suppress tomato disease by inducing the healthy rhizospheric microbiota through alterations to the bulk soil microflora. Plant Soil..

[37] Deng W., Gong J., Peng W., Luan W., Liu Y., Huang H., Mei X., Min Y., Zhu S. (2024). Alleviating soil acidification to suppress Panax notoginseng soil-borne disease by modifying soil properties and the microbiome. Plant Soil.

[38] Xia H., Shen J., Riaz M., Jiang C., Zu C., Jiang C., Liu B. (2024). Effects of Biochar and Straw Amendment on Soil Fertility and Microbial Communities in Paddy Soils. Plants.

[39] Yang Y., Hu J., Wei X., Huang K., Li C., Yang G. (2024). Deciphering core microbiota in rhizosphere soil and roots of healthy and Rhizoctonia solani-infected potato plants from various locations. Front. Microbiol..

[40] Zhou D., Jing T., Chen Y., Wang F., Qi D., Feng R., Xie J., Li H. (2019). Deciphering microbial diversity associated with Fusarium wilt-diseased and disease-free banana rhizosphere soil. BMC Microbiol..

[41] Zhang X., Wang H., Que Y., Yu D., Wang H. (2021). The influence of rhizosphere soil fungal diversity and complex community structure on wheat root rot disease. PeerJ.

[42] Huusko K., Manninen O.H., Myrsky E., Stark S. (2024). Soil fungal and bacterial communities reflect differently tundra vegetation state transitions and soil physico-chemical properties. New Phytol..

[43] Jiao N., Song X., Song R., Yin D., Deng X. (2022). Diversity and structure of the microbial community in rhizosphere soil of *Fritillaria ussuriensis* at different health levels. PeerJ.

[44] Xie Y., Mao J., Wang W., Zhang Z., Zhu J., Gu M., Tang Q., Song S., Huang W., Wang B. (2020). Structures and biodiversity of fungal communities in rhizosphere soil of root rot diseased garlic. Chin. Agric. Sci. Bull..

[45] Song X., Tan J., Li L., Wang Y., Wu X. (2018). Illumina high-throughput sequencing reveals fungal community composition and diversity in root rot of Coptis chinensis in rhizosphere soil. Chin. Tradit. Herb. Drugs.

[46] Chen H., Chen T., Cai L., Li Z., Fang F., Jin S. (2024). Research on soil enzyme activity and fungal community structure in rhizosphere soil of *Actinidia chinensis* Planch with root rot. South China Fruits.

[47] Dong W., Chen J., Liao X., Chen X., Huang L., Huang J., Huang R., Zhong S., Zhang X. (2024). Biodiversity, Distribution and Functional Differences of Fungi in Four Species of Corals from the South China Sea, Elucidated by High-Throughput Sequencing Technology. J. Fungi.

[48] Yang M., Guo H., Duan G., Wang Z., Fan G., Li J. (2024). Role and mechanism of arbuscular mycorrhizal fungi in enhancing plant stress resistance and soil Improvement: A review. China Powder Sci. Technol..

[49] Zeng C. (2016). Study on the Resistance Mechanism of Nine Astragalus Root and Rot and Compre Adsurgens Varieties to Yellow Stunt Hensive Evaluation for Germplasm Characteristics. Ph.D. Thesis.

[50] Ren D., Niu B., Yin Y., Hua Z., Jia S., Gao M. (2022). Isolation, identification and growth characteristics of garlic root rot pathogen Alternaria embellisia DS55-6F. Soil Fertil. Sci. China.

[51] Wang J., Yin M., Duan Y., Wang Y., Ma Y., Wan H., Kang Y., Qi G., Jia Q. (2024). Enhancing water and soil resources utilization via wolfberry–alfalfa intercropping. Plants.

[52] Wang X., Dong Y., Feng G., Yao Q., Liu C., Zhu H. (2024). Differences of soil fungal community structure and driving factors between healthy and mismanaging tea plantations in Heshan of southern China. Acta Microbiol. Sin..

[53] Wang F., Chen Y., Wu Z., Jiang F., Yu W., You Z. (2021). Effects of reduced chemical fertilizer applications on fungal community and functional groups in tea plantation soil. Acta Tea Sin..

[54] Zhang M., Wang N., Zhang J., Hu Y., Cai D., Guo J., Wu D., Sun G. (2019). Soil Physicochemical properties and the rhizosphere soil fungal community in a mulberry (*Morus alba* L.)/alfalfa (*Medicago sativa* L.) intercropping system. Forests.

