# Supplementary material for: Investigating the Rhizosphere Fungal Communities of Healthy and Root-Rot-Infected Lycium barbarum in the Tsaidam Basin, China
Source: Microorganisms. 2024 Nov 28;12(12):2447. doi: 10.3390/microorganisms12122447 (PMC11676810; doi:10.3390/microorganisms12122447)
Supplement: Supplementary file 1 [file microorganisms-12-02447-s001.zip › microorganisms-3328024-supplementary.pdf]

Subsequent Variation Partitioning Analysis (VPA) was utilized to unravel the impact of chemical characteristics on the microbial community structure within the rhizosphere soils of *L. barbarum* (Figure S1). According to the VPA, approximately 25% of the community variation could be ascribed to chemical properties, with pH + SOM, phosphorus, and nitrogen contributing to 5%, 4%, and 3% of the variance, respectively. Moreover, pH and organic matter were identified as the most influential factors shaping the composition of the microbial community.

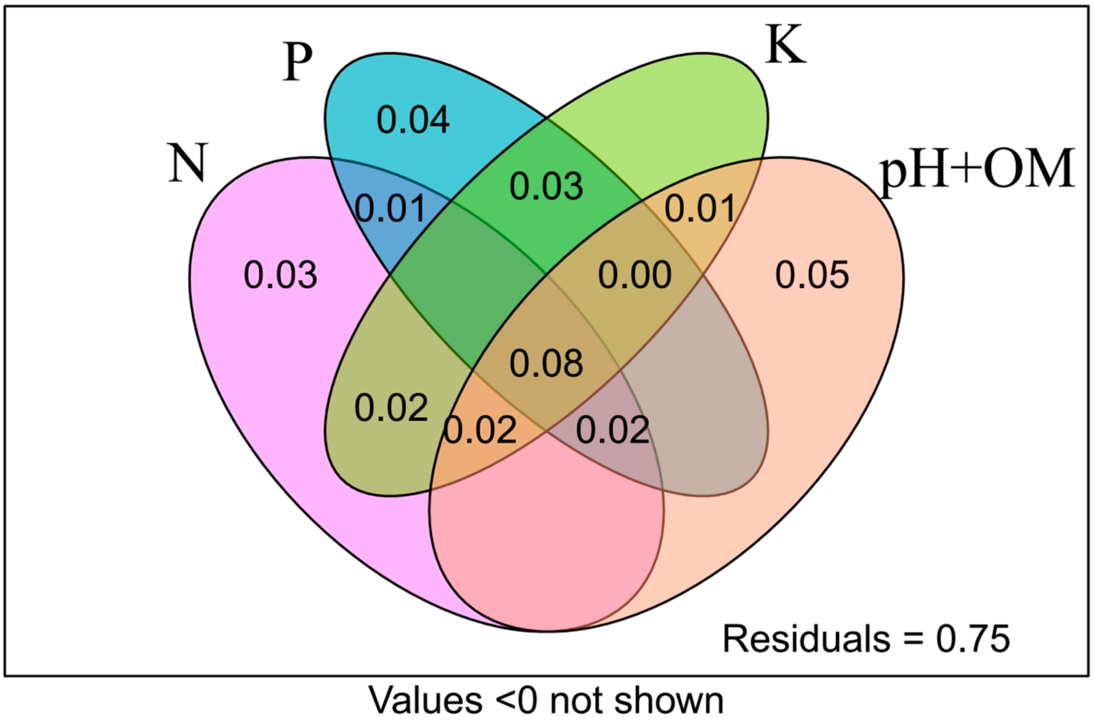

Figure S1: Subsequent Variation Partitioning Analysis (VPA) was utilized to unravel the impact of chemical characteristics on the microbial community structure within the rhizosphere soils of *L. barbarum* (Figure S1). According to the VPA, approximately 25% of the community variation could be ascribed to chemical properties, with pH + SOM, phosphorus, and nitrogen contributing to 5%, 4%, and 3% of the variance, respectively. Moreover, pH and organic matter were identified as the most influential factors shaping the composition of the microbial community.
